# The effect of aquatic and land exercise on the mental well-being of women following breast cancer surgery-comparative study

**DOI:** 10.1007/s10549-023-07088-7

**Published:** 2023-09-04

**Authors:** Ewelina Czenczek-Lewandowska, Ewa Szeliga, Justyna Leszczak

**Affiliations:** Institute of Health Sciences, Medical College, University of Rzeszow, Rzeszow, Poland

**Keywords:** Aquatic physical therapy, Aquatic exercise, Mastectomy, Breast cancer, Physical activity

## Abstract

**Purpose:**

Women after surgical treatment of breast cancer are less likely to engage in physical activity and may exhibit depressive symptoms even for many years post-treatment. The aim of the study was to compare the impact of 6 months aquatic and land exercise on mental well-being of women after breast cancer surgery.

**Methods:**

The study was based on a survey which involved a total of 90 women ≥ 50 years of age (including 60 subjects after treatment of breast cancer ≥ 2 years after surgery and 30 healthy control). The study participants were divided into three groups, i.e. women attending general exercise sessions in a gym (A, *n* = 30), participating in aquatic exercise (B, *n* = 30), as well as healthy female peers reporting no regular physical exercise for the period of 6 months (C, *n* = 30). The present data were acquired using standardized questionnaires designed to assess physical activity (IPAQ-SF), mental well-being (WHO-5) and level of anxiety, i.e., Generalized Anxiety Disorder Questionnaire (GAD-7).

**Results:**

Both the aquatic and land exercise had positive effect on mental well-being of woman after breast cancer surgery and there were no significant differences between them. Analysis of the anxiety level (GAD-7) confirms that women after breast cancer treatment were more exposed to a feeling of generalised anxiety disorder, i.e. 8.67 vs. 6.73, 4.5 (*p* = 0.001). The results of WHO-5 index were significantly higher in the A and B groups comparing to healthy control, i.e. 13.27 vs. 14.18; 10.10 (*p* < 0.001), but the obtained results still indicate the low self-esteem of the study group. Women after breast cancer surgery who regularly participated in aquatic exercise were engaged in more vigorous physical activity compared to the other groups, i.e. 1049.33 vs. 521.33; 860.00 MET min/week (*p* = 0.001).

**Conclusion:**

Regardless of the type of physical activity, 6 months aquatic and land exercise contributed to improved mental well-being and ensured adequate levels of moderate physical activity of woman after BC surgery. Regular physical activity is crucial in the rehabilitation after mastectomy and can be an effective treatment to achieve beneficial mental outcomes.

## Introduction

In 2020 breast cancer (BC) was the most frequently diagnosed cancer in 157 (out of 185) countries in the whole world. Forecasts indicate that by 2040 the number of newly diagnosed BC cases will have increased by over 40% [1–3]. Surgical treatment of BC and its physical and visual consequences are strongly associated with a high mental burden, lower self-esteem and a change in the perception of one’s own body. BC survivors mostly manifest emotional distress, and fear of recurrence of the disease, which may induce typical symptoms of anxiety and depression even in 30–50% of patients [4]. Although the moment of BC diagnosis and early stages of treatment are often the most traumatic and strongly mentally burdensome, the late period after the end of treatment is also accompanied by fear for health, recurrence of the disease, and feeling insecure [5]. Fear after treatment may be equally strong and evaluate in a depression. Many years of observations show that woman after BC surgery have deeper depressive symptoms compared to other types of cancer. In this context, regular physical activity in adequate levels has been considered as a protection factor for the adverse effects on mental health as well as onset and recurrence of cancer [6]. Exercise is necessary element of treatment in comprehensive rehabilitation which should started before the procedure and its constant continuation during the postoperative period [7].

Rehabilitation of women treated for BC is based mainly on exercises in the field of kinesitherapy (individual and group), resistance training programmes, manual lymphatic drainage, self-massage, lymphatic drainage, transcutaneous electrical nerve stimulation (TENS), kinesio taping, intermittent pneumatic compression, bandaging or occupational therapy. In the late period of rehabilitation, group physical rehabilitation is of great importance due to the improvement of the musculoskeletal system as well as social integration, and one of its recommended forms is aquaerobics. An aquatic environment creates unique conditions for therapeutic exercises and gives an opportunity to perform exercises that could not be performed on land [8]. The properties of water, such as its temperature, resistance and hydrostatic pressure, in combination with aerobic, stretching, resistance and stability exercises, carry numerous health benefits, hence this method has been widely used in many areas of rehabilitation including oncological.

The current literature suggest that there is a need to strengthen the level of evidence for this rehabilitative approach to patients following BC surgery. The aim of the study was to compare the impact of 6 months aquatic and land exercise of women after BC surgery on their mental well-being.

## Material and method

### Study design and population

**Fig.1:**
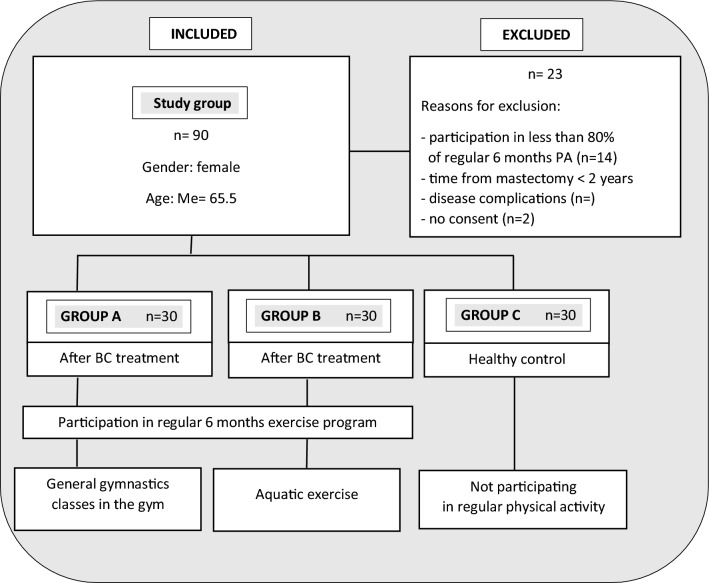
Flow diagram of the study design

The study was designed as a prospective and comparative intervention study. Participants of the study (*n* = 90) were allocated into one of two intervention groups or control group in ratio of 1:1:1. The average age of the women was 66 years (*Me* = 65.5) (Fig. 1).

The studied women were members of the “Association for Women After Mastectomy” (“Amazons”), which organised group rehabilitation (i.e. general classes in the gym and aquatic exercise in the pool). Eligibility criteria included: age above 50 years old, consent of the physician to participate in the group physical exercise, participation in more than 80% of classes in the period of 6 months, informed consent to participate in the study (given before the start), fully completed and returned questionnaire, state after mastectomy minimum 2 years after surgery, no current disease complications, completed adjuvant chemotherapy, radiotherapy and hormonal therapy, controlled blood pressure, member of the Amazons association; lack of reported and diagnosed chronic diseases in the control group. The control group was randomly selected taking into account the gender and age of the participants.

The study was approved by the Ethics Committee of the University of Rzeszow (ref. no 22/10/2020).

#### Interventions

Both forms of intervention, i.e. aquatic and land exercise for woman following BC surgery were held regularly for a period of time once a week, lasting 60 min, and were conducted by a physiotherapist.

All sessions started with a 10-min general cardio warm-up to strengthen the body and concluded with a 10-min cool-down. The initial part of the exercise aims to stimulate the blood circulation, lung function and support the musculoskeletal system to prepare it for the moderate to vigorous exercise in the main part of the training. Exercises engaged all parts of the body (upper limbs, trunk, and lower limbs). Both of the exercise programmers included moderate to vigorous aerobic and resistance training exercise. The final part of the session was based on stretching, calming down and breathing exercises. During aquatic training the participants used noodles, water dumbbells, swimming boards and the water environment was used for resistance exercises. In the land exercise session there were used: rubber small and large sizes, wooden ladders, wooden gymnastic sticks and there band for resistance exercises were used during the performance.

Aquatic exercise was held in a swimming pool measuring 25–12.5 m with a depth of 1.40 m, the water temperature was 25 °C. Participants were given the choice of one kind of the intervention and declared their participation in sessions for the entire semester, which took place regularly for a period of 6 months.

#### Measures

The initial part of the online questionnaire addressed socio-demographic and socio-economic characteristics (such as age, current weight, height, place of residence), history of mastectomy (time after surgery, type of surgery) and the main forms of participation in exercise classes during the last 6 months prior to the study (i.e. general gymnastics classes in the gym, aquaerobics classes, others). The obtained answers allowed for selection of participants for particular groups.

The level of physical activity was determined on the basis of the self-report International Physical Activity Questionnaire-Short Form (IPAQ-SF). Questions can be answered to estimate four intensity levels of physical activity: vigorous physical activity (VPA), moderate-to-vigorous physical activity (MVPA), walking and sedentary behaviour. All activities have been converted to unit values in metabolic equivalent of task (MET min/week). MET is a physiological measure expressing the intensity of physical activities. The task of the participants is to estimate the time they spent in particular activities during the last 7 days of the week (last 7 day recall). IPAQ-SF is a well-known objective and self-reported measure [9].

The WHO (Five) Well-being Index (WHO-Five, WHO-5, or WBI) tool was used to assess mental well-being. The final result of the level of well-being is obtained by adding the results of the answers to five questions (0 to 25 points, where 0 is the lowest and 25 the highest, testifying to the best well-being). This result can be converted to a percentage by multiplying it by 4. Mental well-being was also evaluated using a screening tool used to determine the feelings associated with Generalized Anxiety Disorder 7-item (GAD-7 scale). The questionnaire is used to evaluate a seven items: feeling nervous, anxious, or on edge; being able to stop or control worrying; worrying too much about different things; trouble relaxing; being restless; becoming easily annoyed or irritable and feeling afraid as if something awful might happen. Each item is scored in scale from 0 to 3; total scores ranging from 0 to 21 with higher scores means greater anxiety severity [10].

### Statistical analysis

Statistical analysis of the collected material was performed in the Statistica 13.3 package from StatSoft. A Mann–Whitney *U* test was used to assess differences in the average numerical characteristic level in two populations. The ANOVA Kruskal–Wallis test was used to assess differences in the average numerical characteristic level in more than two populations. The correlation of the two was calculated using the Spearman rank correlation coefficient. The analysis of variables consisting of qualitative data was carried out using Pearson’s Chi-square test. The level of statistical significance was *p* < 0.05.

## Results

### Characteristics of the study groups

The subjects in the three groups did not differ in socio-demographic terms, only the place of residence significantly differentiated the subjects (*p* = 0.003). Amazons in groups A and B more often lived in cities, and women from group C (control), were more often residents of villages (Table 1). The majority of the research group were after radical mastectomy (48%), modified radical mastectomy (35%) and simple mastectomy (17%). The side of the surgery more often affects the left breast (45%), right (42%) or bilateral (13%).

**Table 1:** Characteristics of the study groups

	Group A (*n* = 30)		Group B (*n* = 30)		Group C (*n* = 30)		*p* value
	Mean	± SD	Mean	± SD	Mean	± SD	
Age (years)	66.43	(± 6.59)	67.53	(± 8.18)	63.90	(± 5.05)	0.255
Weight (kg)	75.80	(± 8.3)	73.53	(± 9.72)	72.77	(± 10.18)	0.447
Height (m)	1.63	(± 0.06)	1.63	(± 0.06)	1.64	(± 0.06)	0.352
BMI (kg/m^2^)	28.71	(± 3.28)	27.75	(± 3.08)	26.93	(± 3.67)	0.247
Time after BC treatment (years)	6.43	(± 3.78)	10.13	(± 7.89)	–	–	0.326
Self-reported physical fitness (1–5*)	2.57	(± 1.04)	2.60	(± 0.89)	2.30	(± 0.95)	0.373

	Group A (*n* = 30)		Group B (*n* = 30)		Group C (*n* = 30)		*p* value
Place of residence Urban Rural	*n* 21 9	% 70.0 30.0	*n* 23 7	% 76.7 23.3	*n* 11 19	% 36.7 63.3	0.003*

*BMI* body mass index

*p < 0.05 statistical significance

*1-very good, 2-good, 3-moderate, 4-sufficient, 5-insufficient

Tables 2 and 3 show the results of the three test groups. The results of women in the three groups were not equal on the IPAQ scale (*p* < 0.05) except for sitting time (*p* = 0.589), as well as in WHO-5 (*p* < 0.001) and GAD-7 (*p* = 0.001).

**Table 2:** Physical activity level results

	Group A (*n* = 30)		Group B (*n* = 30)		Group C (*n* = 30)		*p* value
	Mean	± SD	Mean	± SD	Mean	± SD	
Walking (MET min/week)	1563.65	1736.1	1763.85	1736.5	7448.10	3772.7	< 0.001 *A–K *B–K
MVPA (MET min/week)	1301.33	1385.8	950.00	1202.84	590.67	1264.01	0.002 *A–K *B–K
VPA (MET min/week)	521.33	936.4	1049.33	898.78	860.00	2029.8	0.001 *A–B *B–K
Sedentary behaviour (min/day)	193.67	89.15	174.33	95.69	229.33	173.46	0.589
PA total score (MET min/week)	3386.32	2741.7	3763.18	2341.1	8898.77	5341.3	< 0.001 *A–K *B–K

*VPA* vigorous-intensity physical activity, *MVPA* moderate-to-vigorous-physical-activity, *PA* physical activity

*Groups between which statistically significant differences at *p* < 0.05 level were shown in the post hoc test

**Table 3:** Mental well-being results

	Group A (*n* = 30)		Group B (*n* = 30)		Group C (*n* = 30)		*p* value
	Mean	± SD	Mean	± SD	Mean	± SD	
WHO-5 (0–25 pkt.)	13.27	5.26	14.80	4.16	10.10	2.71	< 0.001 *A–K *B–K
WHO-5 (%)	53.07	21.03	59.20	16.62	40.40	10.83	< 0.001 *A–K *B–K
GAD-7 (0–21 pkt.)	8.67	4.81	6.73	4.28	4.50	4.07	0.001 *A–K
GAD-7							
Mild (0–5 points)	7	23.3	10	33.3	21	70.0	< 0.001
Moderate (6–10 points)	9	30.0	16	53.3	8	26.7	
Moderately severe anxiety (11–15 points)	14	46.7	3	10.0	0	0.0	
Severe anxiety (15–21 points)	0	0	1	3.3	1	3.3	

*Groups between which statistically significant differences at *p* < 0.05 level were shown in the post hoc test

### Physical activity of the study groups

On the IPAQ scale, women in group B (participating in aquaerobics) compared to women in groups A and C achieved significantly higher results in the category of vigorous physical activity (VPA) (*p* = 0.001).

Women in groups A and B achieved significantly higher results in the moderate to vigorous level of physical activity (MVPA) (*p* = 0.002) compared to women in group C.

Women in group C achieved significantly higher results compared to women in groups A and B, in the walking category (*p* = < 0.001) and in terms of overall physical activity (< 0.001). All differences were found to be statistically significant (Table 2).

### Mental well-being of the study groups

On the WHO-5 scale, women in groups A and B achieved a significantly higher well-being index than women in the C-control group, which proves better mental well-being compared to the control group (*p* < 0.001). However, there were no differences between groups A and B.

At the same time, on the GAD-7 scale, women in groups A and B achieved significantly higher results than women in the C-control group (*p* = 0.001). However, there were no differences between groups A and B (Table 3).

### Selected characteristics of the participants and results of the WHO-5, IPAQ and GAD-7 scales

Of the women in group A, the younger they were and the better they assessed their physical fitness, the higher the score in the WHO-5 scale (*p* = 0.012; *p* = 0.013).

In the C-control group, the higher the BMI the respondents had, the higher the total score in the IPAQ scale (*p* = 0.030).

In group B, the shorter the time since the woman’s operation, the more points they scored in the WHO-5 scale (*p* = 0.042), the better they assessed their physical fitness, and the higher the results on the IPAQ scale in terms of overall activity level (*p* = 0.004) (Table 4).

**Table 4:** Analysis of selected factors and the results of the scales

Characteristic	Scale	Group A (*n* = 30)		Group B (*n* = 30)		Group C (*n* = 30)	
		R Spearmana	p	R Spearmana	p	R Spearmana	p
Age	WHO-5	− 0.45*	0.012*	− 0.19	0.311	− 0.08	0.672
	IPAQ total score	− 0.08	0.692	− 0.26	0.158	− 0.06	0.752
	GAD-7	0.14	0.453	0.03	0.874	− 0.15	0.419
BMI	WHO-5	− 0.13	0.500	− 0.06	0.735	0.22	0.253
	IPAQ total score	− 0.28	0.134	− 0.02	0.923	0.40*	0.030*
	GAD	0.06	0.764	− 0.05	0.795	− 0.18	0.340
Time after BC treatment	WHO-5	− 0.16	0.410	− 0.37*	0.042*	–	–
	IPAQ total score	− 0.04	0.818	− 0.12	0.515	–	–
	GAD-7	− 0.11	0.549	− 0.03	0.893	–	–
Self-reported physical fitness	WHO-5	− 0.45*	0.013*	− 0.26	0.165	− 0.33	0.078
	IPAQ total score	− 0.34	0.067	− 0.51*	0.004*	− 0.16	0.398
	GAD-7	− 0.29	0.123	0.16	0.406	0.02	0.905

*Groups between which statistically significant differences at *p* < 0.05 level were shown in the post hoc test

The place of residence did not affect the results obtained by the respondents in the WHO and GAD scales in any of the groups (*p* > 0.05).

## Discussion

Women with BC can present reduced activity as well as depressive symptoms even many years after treatment. High levels of physical activity and mental well-being are crucial for achieving better treatment results and disease prognosis as well as prevention of its recurrence. Pre-diagnosis and post-diagnosis PA are associated with a reduction in the risk of both BC-specific mortality and all-cause mortality [11, 12].

The presented results confirm that every kind of the physical activity, i.e. aquatic or land exercise are meaningful in the comprehensive process of improving the mental and physical state of women after mastectomy. The results of our own research confirmed that a 6-month regular exercise programme mostly has helped to maintain higher level of physical activity undertaken in the degree of VPA and MVPA and mental well-being in comparison with heathy control who didn’t engage regularly in physical activity.

### The importance of physical activity after mastectomy

Physical exercises are the best non-pharmacological treatment for several side effects of cancer-related therapies and they play a beneficial role in every stage of treatment of oncological disease. According to ASC guidelines (2022) physical exercise has a beneficial effect in reducing upper limb volume, improving upper body strength, and increasing muscle mass and cardiorespiratory fitness, reducing body fat and waist and hip circumference. Much more current studies discover strong associations of physical activity with psychological and emotional benefits. Physical and psychological factors are equally important for the long-term outcomes of BC [13].

Every woman after BC treatment should engage in regular physical activity, and to be precise, they should spend a minimum of 150 min on moderate-to-vigorous physical activity (MVPA) per week or 75 min of vigorous physical activity (VPA) each week, even during adjuvant cancer treatment such as radiation or chemotherapy [14]. These recommendations are consistent with WHO Guidelines (2020) on physical activity and secondary behaviour for older adults (aged 65 years and older) including those with chronic conditions and those living with disability. The importance of varied multicomponent physical activity that emphasises functional balance and strength training at moderate or greater intensity on 3 or more days a week is stressed [15].

The recommended forms of physical activity after BC removal include Nordic walking [16], dance therapy [17], music therapy, virtual reality therapy [18], yoga [19], Tai Chi [20], reflexology as well as aquatic physical therapy [21]. Women who are involved in vigorous physical activity minimize the risk of BC up to 30–40% [22].

### Aquatherapy in improving the physical fitness of women with BC

Aquatic physical therapy—also known as hydrotherapy or aquatic exercise has been used in many areas of rehabilitation, including in improving the physical fitness of patients after cancer treatment, mostly diagnosed with BC [23].

The effect of an aquatic environment on the functions of the body are well known. Water buoyancy reduces the feeling of gravity, relieving the movement organs including the spine and peripheral joints. Buoyancy forces promote the feeling of better physical fitness and weight loss, improving mental well-being. Water creates favourable conditions for performing effective resistance exercises that increase muscle strength and endurance, as well as stretching exercises that make muscles more elastic and support the improvement of mobility in the peripheral joints. In addition to improving physical fitness, exercises in water counteract the formation of chronic arm oedema, which are a common complication (amongst 1 in 5 patients) after BC treatment [24].

Despite the many well-known health benefits of aquatherapy, the results of global research are divergent, depending on the parameters analysed. Reger et al. also highlight differences in methodology and extremely heterogeneous results of the current studies about the effectiveness of aquatic exercise on cancer patients [25]. Meta-analyses by Marchica et al. and Yeung W, show that there were no significant differences between the effect of aquatic therapy compared to conventional physical exercise and standard care. The authors analyze lymphedema surgery status, volumetric lib change and physical function of the upper limbs [26]. The meta-analysis of Mur-Gimeno shows that moderate to large improvements were found after aquatic therapy compared to typical physical exercise on land. The most significant effect has been found in pain, shoulder range of motion, pulmonary function, HRQoL, cardiorespiratory fitness and muscle strength. There were no differences in the effect on fatigue, lymphedema and body mass composition [20].

The literature shows that the most optimal period for participation in aquatic exercise is 4–6 months. For the elderly, 4 months (16 weeks) of aquatic exercises, can significantly improve lower and upper body strength as well as flexibility, functional mobility and balance [27]. In our own study, a period of 6 months of regular aquatic physical therapy after BC surgery had a positive effect on the higher amount of active time spent in VPA and MVPA, which are crucial for maintaining physical fitness.

On the IPAQ scale, women who took part in aquatic exercise (group B) undertook significantly more vigorous physical activity (VPA), compared with women exercising in the gym (528 MET min/week more) and women in the control group (189.33 MET min/week more) (*p* = 0.001). Similarly in the case of MVPA, women after mastectomy (both groups A and B) achieved better results than the control group (group C) (710.6 MET min/week and 359.33 MET min/week more) (*p* = 0.002).

Any form of regular, group physical activity is an opportunity to exercise with vigorous or moderate intensity, which are the most beneficial for health. In this case, the disease, as well as the treatment and participation in classes organised by the Amazons Association had a positive effect on health behaviours related to physical activity. They were much better than those of the healthy women. Women in the control group (group C) achieved higher results in the walking category (*p* = < 0.001). Although they were less likely to participate in organised exercise groups, during the day they were active for longer, at light physical activity (LPA) level, and in terms of overall physical activity they achieved higher results (< 0.001) [28]. Kim et al. showed a reduced mortality risk in individuals with LPA than in those with totally sedentary behaviour [29].

The studied groups did not differ significantly in the amount of time spent sitting (on average 199 min a day, about 3 h). According to WHO guidelines (2020) replacing sedentary activities with any intensity of physical activity (including light intensity) has health benefits [14].

### Aquatherapy and mental well-being

The diagnosis of BC as well as the implementation of oncological treatment significantly burden the psychological well-being of a woman increasing the risk of more serious mental illnesses. Changes in body appearance after necessary surgical treatment and physical problems like fatigue, nausea, alopecia, decreased aerobic capacity and loss of muscle strength are traumatic experience for the woman [30]. The most frequent mental problems observed in the patients after BC surgery are depression and anxiety disorders but also sleep disorders, fatigue syndrome and decreased quality of life [31]. This group is exposed to depressive symptoms 13–18% more often than the general population [32].

The period of diagnosis and early treatment is the most difficult for the patient’s psyche, but during this period the woman is monitored medically by a team of specialists. The late stage after the surgery is also associated with numerous mental burdens, hence the continuation of multidimensional care for the woman is important. An important role in this period is played by peer-support organizations—Amazons Clubs, which provide meetings with specialists as well as other women with the same experiences, which is good form of therapy and prevention of mental problems [33].

Supportive and forms of therapy is particularly important in oncology treatment and minimizing the side effects. A healthy lifestyle that includes regular physical activity is essential to recovery and it is closely related to the support of mental health and learning how to live after the oncological treatment [34]. There are no official guidelines on the psychological treatment of women after BC. Whereas there is moderate-certainty to high-certainty evidence that PA can decreased symptoms of depression and anxiety [14]. Aquatic exercise can result in positive mood changes which are effect of activation of parasympathetic nervous system and increases in plasma dopamine levels [35, 36].

In our study, on the WHO-5 scale, women in groups A and B achieved a significantly higher Well-being Index than women in the C-control group, which proves their better mental well-being compared to the control group (*p* < 0.001). There were no differences between groups A and B, so the type and form of exercise classes was not important. Although the results of groups A and B were higher, the average score was only 14 points out of the possible 25 (56%). The interpretation of the results suggests a need to deepen the test for the presence of depression if the raw score is less than 13, so the result obtained was close to this limit.

The analysis of the GAD-7 results indicates that women in groups A and B achieved significantly higher results than women in the C-control group (*p* = 0.001), so they were more exposed to a feeling of generalised anxiety. This group especially should be under the care of psychologists in order to diagnose the onset of mental illnesses such as depression or generalised anxiety. Similar to the WHO-5 study, no differences were found between groups A and B (Table 3).

Despite the results obtained, most scientific reports clearly emphasise the beneficial effect of physical activity on mental well-being as well as the treatment of mental illnesses. The latest and extensive meta-analysis of the subject was done by Wang et al. Based on the available literature (five studies comprising 356 participants), they conclude that aquatic physical therapy is a valuable method of improvement after BC treatment, and its greatest effects were seen in the field of improvement of fatigue and quality of life. The buoyancy force of the water reduces the stress on painful weight-bearing joints which helps relieve fatigue [37]. Similar conclusions are drawn by Cantarero et al., who observed a positive impact of a 2-month aquatic programme on cancer-related fatigue, tension, depression, anger and mental fatigue [38]. In turn, Odynets et al. confirm that 12 months of aquatic intervention improved the emotional well-being of women after BC treatment and was a more effective form of activity compared to Pilates and yoga exercise [39].

### Limitations

The WHO-5 and GAD-7 tools are not diagnostic tools and their results are not a diagnosis of mental disorders. They are used for the preliminary diagnosis of mental illnesses such as depression or generalised anxiety syndrome. Questionnaires are subjective and highly dependent on individual perception. The composition of the control group, consisting of women without a breast cancer diagnosis, also presents a limitation. Responses from groups A and B may hold more significance, as they have greater potential for improvement, whereas the control group’s responses may not exhibit the same degree of change.

## Conclusions

1. Regardless of the type of physical activity, 6 months aquatic and land exercise contributed to improved mental well-being and ensured adequate levels of moderate physical activity of woman after BC surgery.
2. Despite of the low mental well-being results of woman after BC surgery, they were higher than in the control group who did not participate in any kind of organized physical activity.
3. Regular physical activity is crucial in the rehabilitation after mastectomy and can be an effective treatment to achieve beneficial mental outcomes.

## Data Availability

The datasets generated during and/or analyzed during the current study are available from the corresponding author on reasonable request.
